# Hyaluronan Synthase 3 Null Mice Exhibit Decreased Intestinal Inflammation and Tissue Damage in the DSS-Induced Colitis Model

**DOI:** 10.1155/2015/745237

**Published:** 2015-09-10

**Authors:** Sean P. Kessler, Dana R. Obery, Carol de la Motte

**Affiliations:** Department of Pathobiology, Lerner Research Institute Cleveland Clinic, 9500 Euclid Avenue, Cleveland, OH 44195, USA

## Abstract

Hyaluronan (HA) overproduction is a hallmark of multiple inflammatory diseases, including inflammatory bowel disease (IBD). Hyaluronan can act as a leukocyte recruitment molecule and in the most common mouse model of intestinal inflammation, the chemically induced dextran sodium sulfate (DSS) experimental colitis model, we previously determined that changes in colon distribution of HA occur before inflammation. Therefore, we hypothesized that, during a pathologic challenge, HA promotes inflammation. In this study, we tested the progression of inflammation in mice null for the hyaluronan synthase genes (HAS1, HAS3, or both HAS1 and HAS3) in the DSS-colitis model. Our data demonstrate that both the HAS1/HAS3 double and the HAS3 null mice are protected from colitis, compared to wild-type and HAS1 null mice, as determined by measurement of weight loss, disease activity, serum IL-6 levels, histologic scoring, and immunohistochemistry. Most notable is the dramatic increase in submucosal microvasculature, hyaluronan deposition, and leukocyte infiltration in the inflamed colon tissue of wild-type and HAS1 null mice. Our data suggest, HAS3 plays a crucial role in driving gut inflammation. Developing a temporary targeted therapeutic intervention of HAS3 expression or function in the microcirculation may emerge as a desirable strategy toward tempering colitis in patients undergoing flares of IBD.

## 1. Introduction

HA is a ubiquitous carbohydrate polymer produced by a wide variety of cell types and is present in the extracellular matrix of healthy and inflamed tissues. This glycosaminoglycan is produced as a long, straight chain polymer of up to 10^4^ disaccharide units of alternating N-acetyl glucosamine and glucuronic acid without a protein core or any postsynthesis decoration or chemical modification [1]. Regulated hyaluronan production occurs at the cytoplasmic membrane surface by one or more hyaluronan synthase (HAS1, HAS2, or HAS3) enzymes before being extruded into the extracellular matrix as a space-filling, supportive anionic molecule [2]. Temporally and spatially controlled production of hyaluronan is critical in heart development as supported by the observation that the HAS2 null mutation in mice is embryonically lethal due to malformation of the heart tissue [3] and HAS3 null mice display altered neuronal activity and seizures [4]. HAS2 production of hyaluronan in skin fibroblasts is important for protecting skin from environmental stress that can induce apoptosis [5, 6]. Hyaluronan is found in almost every tissue and is especially abundant in the vitreous of the eye [7], synovial fluid in joints [8], proximal tubules of the kidney [9], the mucosal layer of the colon [10, 11], and vertebrate breast milk [12]. HA is even present in platelets and their megakaryocyte precursors; however, in these cells it accumulates in the intracellular space [13]. Hyaluronan degradation and cellular turnover are governed largely by the hyaluronidase family of enzymes, (e.g., HYAL 1 and HYAL 2) in a CD44-dependent [14] or, as recently described in a KIA1199 protein dependent, CD44-independent fashion [15].

Our lab has been investigating the role of HA in intestinal inflammation, especially as it relates to IBD. IBD encompasses human diseases Crohn's colitis and ulcerative colitis, conditions that exhibit increased HA deposition during flares, or periods of inflammation [10]. In addition, we have shown that viral infections, ER stress, and inflammatory cytokines (especially the IBD associated factor, TNF*α*) cause formation of an HA rich leukocyte adhesive matrix around intestinal smooth muscle cells [10, 16] and on intestinal microvascular endothelial cells [11]. This HA matrix binds mononuclear leukocytes, including monocytes and lymphocytes and potentially contributes to inflammatory infiltrate recruitment during colitis [17].

The experimental DSS-induced murine colitis model is a well-established and consistent method to study profound inflammatory changes that occur in the gut during disease progression [18]. We previously demonstrated that hyaluronan deposition precedes inflammation and immune cell influx into damaged gut tissue in this model [11]. In addition, we observed that leukocyte adhesive, cable-like hyaluronan structures are produced at the microvessel surface and into the lumen of DSS-treated mice. Similar HA structures are observed in the microvasculature of colon tissue sections derived from IBD patients and importantly on* in vitro* grown human intestinal microvessel endothelial cells (HIMECs) isolated from colon when treated with the inflammatory cytokine, TNF*α* [13]. Interestingly, we discovered that, exclusively, HAS3 mRNA expression increased in HIMECs after TNF*α* exposure, suggesting that HAS3 is the enzyme chiefly responsible for generating luminal HA during disease [13]. Proinflammatory leukocytes [17] and platelets [13] home on these cable structures, degrading them into smaller-size proinflammatory signaling molecules which may perpetuate the inflammation cycle [13]. Based on these observations, we have proposed that increased production of HA during pathologic events, particularly HA in the microvasculature produced by HAS3, may be a key player in driving the unending cycle of inflammation experienced by IBD patients [13].

We experimentally tested whether disrupting HA production affects the inflammation process that occurs in the murine DSS-colitis model, using HAS1 null, HAS3 null, and HAS1/HAS3 double null mice. We compared the disease progression and histological changes in the colon over the ten-day time course of disease and found that mice that carried the HAS3 null mutation had much less severe colitis by every means of analysis. Weight loss, disease activity, colitis scoring, circulating IL-6 levels, and histology/immunostaining were all ameliorated in HAS3 null mice experiencing colitis. Our data suggest that controlling HAS3 expression in the microvasculature of IBD patients may be a significant target site for therapeutic drug intervention.

## 2. Materials and Methods

### 2.1. Mice

All experiments were performed under an animal welfare protocol approved by the Lerner Research Institute's Institutional Animal Care and Use Committee (IACUC). All mice were at adult age and c57Bl/6 background strain housed and bred in specific pathogen-free (SPF) microisolator cages on standard Teklad irradiated chow in the AALAC certified LRI Biological Resource Unit and acidified water. Wild-type mice (c57Bl/6J Stock number 000664) used for experiments and breeding with HAS1/3 double null mice were purchased from Jackson Labs, Bar Harbor, ME. HAS1/HAS3 double null mice, created by crossing single null mice [19, 20], were a generous gift from Dr. Vincent Hascall in the Biomedical Engineering Department at the Lerner Research Institute [5]. HAS1KO and HAS3KO single null mice were generated by breeding the double null mice with wild-type c57Bl/6J mice and then interbreeding for the desired null allele from a dihybrid cross. Genotypes were confirmed by PCR using Qiagen (Alameda, CA) prepared genomic DNA and an Epicentre Technologies (Madison, WI) PCR Kit in a Thermo Scientific HyBaid PCR Express machine (Wilmington, DE) with the following run parameters: 95°C for 30 sec, 65°C for 30 sec, and 72°C for 1 min, 35 cycles. The amplified PCR products generated by each primer set are as follows: 341 bp HAS1 wild-type allele (mHAS1s: 5′gacgttctggccctggtcctac 3′ and mHAS1as: 5′gggctctactgctgcttggagg 3′) and 320 bp HAS3 wild-type allele (mHAS3s: 5′ggaagcaggcataggtagccttg 3′ and mHAS3as: 5′tgatcggcaccttaccaaccgag 3′). The null allele specific antisense primer (PGKpromAS-1: 5′gaggccacttgtgtagcgccaag 3′) was used with the sense primers above to verify single (HAS1KO 331 bp, HAS3KO 320 bp) and double null mouse genotypes. Adult mice of the same sex and genotype were cohoused in groups of not more than 5 mice per SPF microisolator cage in the LRI Biological Resource Unit with 12-hour light/dark cycles and constantly monitored temperature and humidity conditions with husbandry performed by the LRI BRU staff.

### 2.2. DSS Colitis

The dextran sodium sulfate experimental mouse model was performed as previously described [11]. Briefly, mice received irradiated Harlan Teklad standard chow and acidified in-house water ad libitum in water bottles without or with the addition of 2.5% dextran sodium sulfate (number 160110) (MP Biomedicals, Solon, OH). Mice were weighed and monitored daily for signs of colitis. Mice (*n* ≥ 6 per genotype) were sacrificed following IACUC approved methods on days 0, 3, 5, 7, and 10. Serum was collected by cardiac bleeding. Colons were removed, measured for length (cm) from the rectum to the cecum, and then fixed in ten times tissue volume of molecular biology grade Histochoice (AMRESCO, Solon, OH) prior to processing by LRI Histology Core Service Department.

### 2.3. Disease Activity Index Scoring

During the administration of DSS, all mice were observed for outward signs of colitis using a scoring system based on previous studies [21]. Points were recorded according to the following scale with minimum of 0 points and maximum total of 11 points. Weight change was calculated as (current day weight − starting weight)/starting weight × 100: (0) 0–5%, (1) 6–10%, (2) 11–15%, (3) 15–20%, and (4) >20%. Posture was observed as (0) normal, (1) hunched. Coat fur was observed as (0) normal, (1) ruffled. Stool texture and consistency was observed as (0) normal, (1) soft, (2) soft with blood, and (3) liquid, bloody. Rectal prolapse was observed as (0) none, (1) 1 mm, and (2) 2 mm.

### 2.4. Colitis Scoring

On the day of sacrifice (day 0, 3, 5, 7, and 10), 1 cm rectal sections from all mice were removed and fixed in Histochoice for 24 hours at 25°C. Subsequently, tissue was processed and cut at 2.5 um cross-sections before staining with hematoxylin and eosin by the Lerner Research Institute Histology Core. Rectal sections from all mice were scored based on previous rating systems with a minimum score of 5 and a maximum score of 20 [22, 23]. Epithelium integrity was observed as (1) intact and well-defined crypt structure, (2) intact epithelium with reduced crypt structure, (3) breaks in epithelium layer and reduced crypt structure, and (4) loss of epithelium and crypt structure. Infiltration of leukocytes was observed as (1) none, (2) sparse, (3) moderate, and (4) fulminant. Submucosal swelling below the muscularis mucosae was observed as (1) none, (2) minor, (3) moderate, and (4) severe. Hyperplasia of the muscularis mucosa was observed as (1) none, (2) minor, (3) moderate, and (4) severe. Changes in angiogenesis were recorded, noting an increase in the number, size, and thickness of blood vessels during colitis: (1) none, (2) minor, (3) moderate, and (4) severe.

### 2.5. Histology and Immunohistochemistry of Rectal Sections

Hematoxylin and eosin stained rectal section images were captured with a 10x objective lens on a Leica Digital Microscope using Leica DFC425C camera and Leica Application Suite software (Leica Corp, Buffalo Grove, IL). Disease-associated changes in hyaluronan abundance and location were observed by immunostaining following previously published methods [24]. Briefly, rectal sections were deparaffinized by repeated dipping in the following solutions: Clear-Rite 3 (2 × 3 min), Flex 100 (2 min, 1 min), Flex 95 (2 min, 1 min) (Richard Allan-Thermo Scientific, Kalamazoo, MI), and tap water. Tissue was encircled with a Pap-Pen (Research Products International, Mt. Prospect, IL) before blocking for 30 min in 2% Fetal Bovine Serum-Hank's buffered saline (2% FBS-HBSS). The biotinylated hyaluronan binding protein (HABP) (Calbiochem-EMD Millipore, Billerica, MA) was diluted (1 : 100) in 2% FBS-HBSS and applied overnight at 4°C. Subsequently, slides were washed 3 times in HBSS before applying streptavidin-488 (1 : 500) (Life Technologies, Grand Island, NY) in 2% FBS-HBSS for 45 minutes in the dark at 25°C. Slides were washed 3 times in HBSS prior to mounting under cover glass with Vectashield + DAPI (Vector Labs, Burlingame, CA) and sealing with nail polish. Stained tissues were imaged at 10x magnification with a Leica microscope using ImageProPlus and Photoshop software. Scale bars = 200 um. Semiquantitative measurement of submucosal swelling in the histology images and semiquantitative densitometry analysis of HA deposition in the immunohistochemistry images were performed with ImageProPlus software.

### 2.6. Serum IL-6

Serum was collected from all mice in Microtainer serum separator tubes (Becton Dickenson, Franklin Lakes, NJ), centrifuged at 4100 rpm and stored at −80°C. Twenty microliters of serum were tested in duplicate at all time points in the BioLegend Mouse IL-6 ELISA Max platform (BioLegend, San Diego, CA) according to manufacturer's instructions. The serum was also measured for protein concentration using the Bio-Rad Bradford assay system (Bio-Rad, Hercules, CA). Both assay plates were read with the SpectraMax 340PC384 (Molecular Devices Corp., Sunnyvale, CA). The concentration of IL-6 (ng/uL) was then normalized to total protein in the serum (ug/uL) to determine the ng IL-6/ug total protein using Microsoft Office 2010 Excel followed by graphing with GraphPad Prism 5 software.

### 2.7. Statistics

All data was analyzed with GraphPad Prism 5 software. The data collected from each group of mice of the same genotype at the same time point was plotted as an average with error bars signifying the standard error of the mean (SEM). A one-tailed unpaired student *t*-test or a Mann-Whitney *t*-test for nonparametric data at 95% confidence was then applied to compare a mutant strain to wild-type strain for the indicated parameter or a one-factor ANOVA test of significance was applied when comparing multiple groups to each other.

## 3. Results

**Figure 1:**
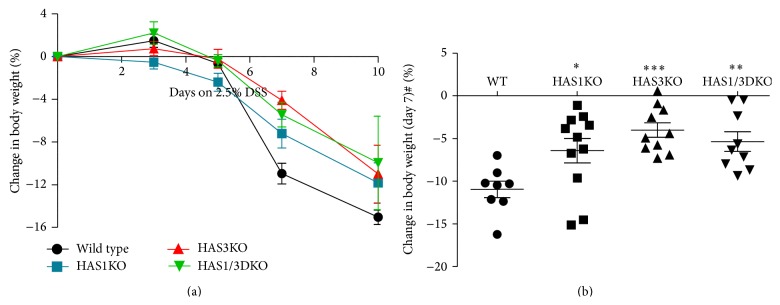
Change in body weight during DSS-induced colitis. (a) Groups of 6 or more adult mice of each indicated genotype were provided with 2.5% DSS in their drinking water for up to 10 days. Body weight measured at days 3, 5, 7, and 10 was compared to starting weight at day 0 for each mouse. The data points reflect the average change in body weight with error bars representing ±SEM. (b) Significance of day 7 weight loss of genotypes compared to the wild-type group: ^*∗∗∗*^ *P* < 0.0001, ^*∗∗*^ *P* < 0.005, and  ^*∗*^ *P* < 0.05 in Student's unpaired *t*-test, 95% confidence. One-way ANOVA test for the mean weight loss compared for all groups ^#^ *P* < 0.005.

To test our hypothesis that HA is a key molecule that drives inflammation in the gut during intestinal inflammation, we treated mice that were null for either HAS1 or HAS3 or both HAS1/HAS3 in the experimental DSS-colitis model. Weight loss is a reliable and accepted measure of disease activity in this model [22, 25].  Figure 1 illustrates that all genotypes, except the HAS1 null mice, gain a small amount (1–3%) of body weight during the first three days of DSS treatment. Continued exposure to DSS, however, causes dramatic weight loss in the wild-type group between day 5 and day 7 but is less severe in the three HAS null groups, on these days. While all three null groups were protected from weight loss at all time points compared to wild-type mice, the HAS3 null group lost significantly (*P* < 0.0001) less body weight by day 7 (Figure 1(b)). Although the mean weight loss measured between HAS3 and HA1 null groups is notable, it approached but did not fully reach statistical significance at day 7 time point. HAS3 null mice lost an average of less than 5% bodyweight compared to the average loss of 11% by the wild-type group. We focused on day 7 time point because this is the time point where the most dramatic changes in disease progression occur, although the animals continue to lose weight through day 10 and are variably distressed.

**Figure 2:**
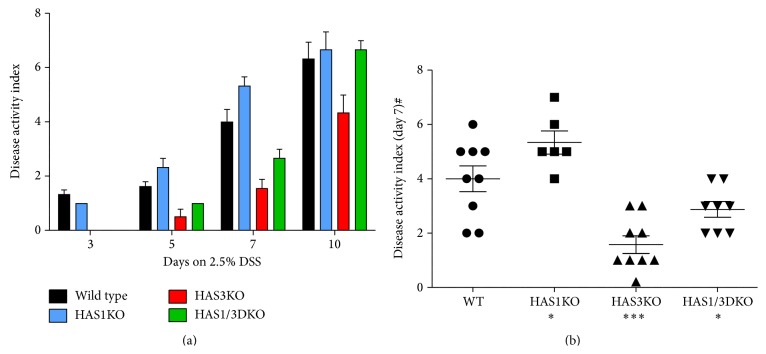
Disease activity index (DAI) during DSS-induced colitis. (a) All mice were observed for outward signs of disease including weight loss, hunched posture, ruffled fur, bloody stools, and rectum prolapse. Data bars reflect the average of six or more mice per time point for each genotype with error bars set at the ±SEM. (b) DAI at day 7. Significance of day 7 weight loss of genotypes compared to the wild-type groups: ^*∗∗∗*^ *P* < 0.002 and ^*∗*^ *P* < 0.05 in a Mann-Whitney nonparametric unpaired *t*-test, 95% confidence.

**Figure 3:**
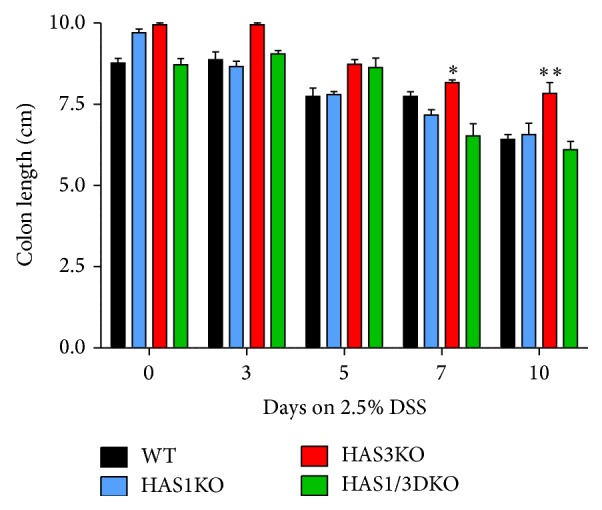
Decrease in colon length of DSS-treated mice. Colons removed from mice sacrificed at the indicated time points were measured for distance (cm) from the distal end of the cecum to the rectum. Bars represent the average of 6 or more mice from each indicated genotype at the noted time point. Error bars reflect the ±SEM. The significant decrease of colon length at day 7 for the HAS3 null mice is indicated; ^*∗*^ *P* < 0.05, ^*∗∗*^ *P* < 0.01.

In addition to weight loss, 2.5% DSS-treated c57B/6 background strain wild-type mice typically exhibit rectal bleeding between day 5 and day 7 with stools becoming soft and loose to the point of being liquid by day 10. At day 10, mouse fur can take on a ruffled appearance as the mice display reduced motility and adopt a hunched posture. We therefore noted this disease activity (DAI) by assigning points by following a previously reported scoring index as described in the methods section, based on weight loss, ruffled fur, hunched posture, rectal bleeding, bloody stools, and rectal prolapse [21]. Our data revealed that the HAS3 null mice displayed the lowest disease activity score at all time points (Figure 2(a)) compared to the other three groups. Comparing the DAI at day 7, we observed that HAS1 mice were more severely affected compared to wild-type mice (*P* < 0.05) while both HAS3 and HAS1/HAS3 null mice were significantly more protected (*P* < 0.002 and *P* < 0.05, resp.) (Figure 2(b)). Another associated marker of colitis scoring for this model is colon shortening. During the progression of disease, overall colon length shortens from 9.5–10 cm in untreated mice to approximately 5.5–6 cm in wild-type mice treated with DSS for 10 days.  Figure 3 illustrates the shortening of the colon in all genotypes over the course of the DSS treatment with HAS3 null mice retaining the most colon length at day 7 (*P* < 0.05) and day 10 (*P* < 0.001) time points, respectively.

**Figure 4:**
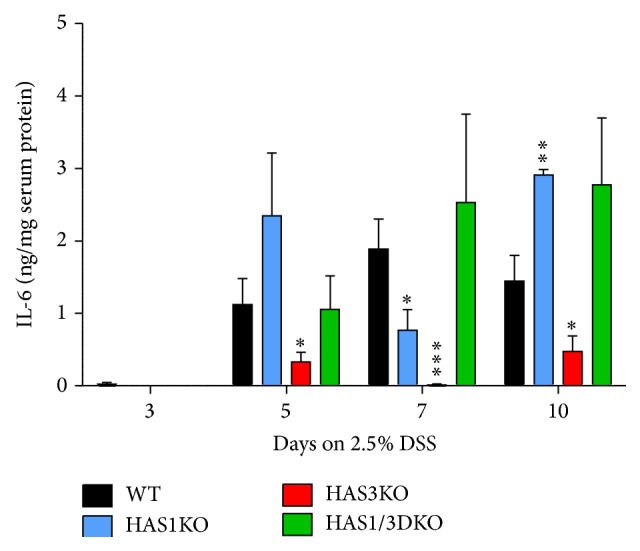
Serum IL-6 in DSS-treated mice. Serum isolated from all mice was tested for IL-6 in an ELISA format, normalized by protein concentration in the serum sample by Bradford assay to determine the ng IL-6/ug total serum protein. Bars represent the average of mice of the same genotype at the indicated time point. Error bars reflect ±SEM. Comparison of null mice to wild-type mice at the indicated time point: ^*∗*^ *P* < 0.05, ^*∗∗*^ *P* < 0.01, and ^*∗∗∗*^ *P* < 0.005 at 95% confidence.

Elevated levels of the inflammatory cytokine IL-6 have been documented in IBD patients and in experimental mice treated with DSS [26, 27]. To test whether the decreased DAI and retention of body weight observed in HAS3 null mice were also accompanied by a change in circulating IL-6 levels, we collected serum from all mice on the day of sacrifice by cardiac puncture. Serum IL-6 levels, measured in an ELISA format and normalized for total protein concentration, are presented in Figure 4. We observed that the HAS3 null mice possessed the lowest amount of circulating IL-6 at all time points we tested, but especially at day 7, compared to wild-type mice (*P* < 0.005). At day 7, IL-6 levels in the HAS3 null mice were barely detectable, whereas wild-type, HAS1 null, and HAS1/3 double null mice all have elevated circulating IL-6 in the 1.0–2.5 ng/mg range. Interestingly, the HAS1/HAS3 null mice had the highest level of IL-6, even surpassing levels found in wild-type mice at each time point.

**Figure 5:**
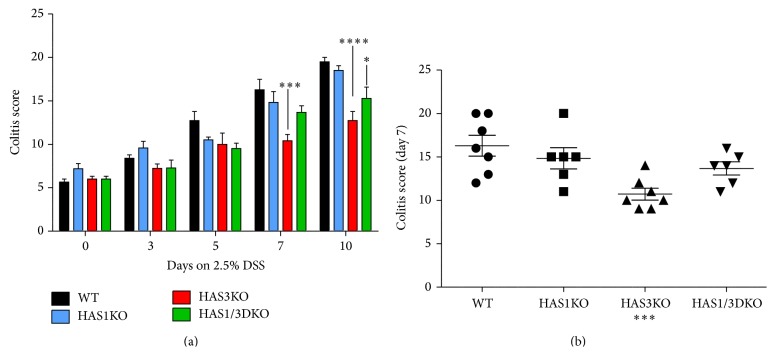
Histological colitis scores for DSS-treated mice. Hematoxylin and eosin stained rectal cross-sections from DSS-treated mice were scored for pathology changes including erosion of the epithelium layer, leukocyte infiltration, submucosal swelling, muscularis mucosae hyperplasia, and increased vascularization as described in methods. (a) Bars represent the average scores of groups of mice of the same genotype at the indicated time points with error bars reflecting ±SEM. Comparison of null mice to wild-type mice at the indicated time point: ^*∗*^ *P* < 0.05, ^*∗∗∗*^ *P* < 0.005, and ^*∗∗∗∗*^ *P* < 0.001 at 95% confidence. (b) The colitis score at day 7 is presented with significance of HAS3 null compared to wild-type mice at 95% confidence noted ^*∗∗∗*^ *P* < 0.005.

**Figure 6:**
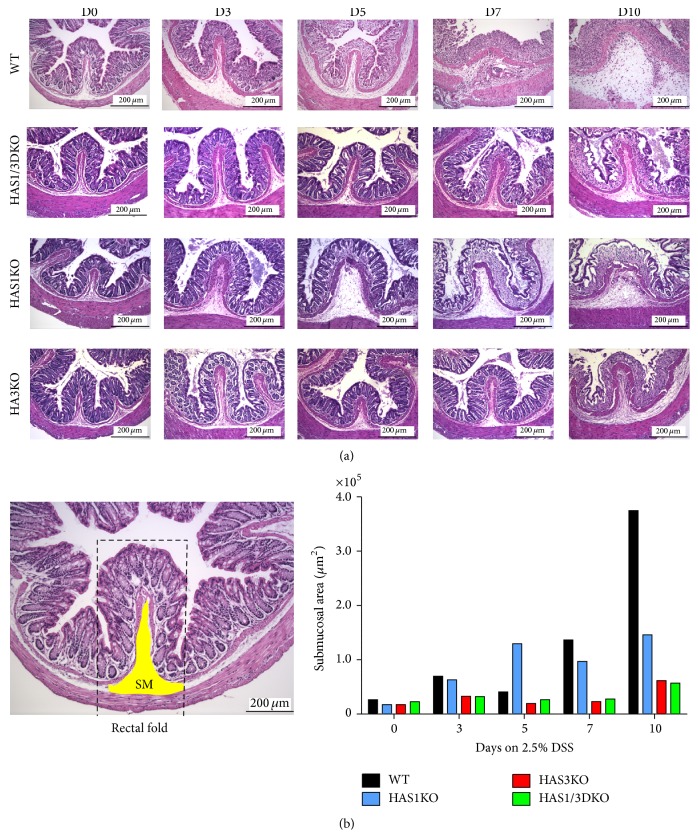
Histology of rectal sections from DSS-treated mice. (a) Representative rectal images of hematoxylin and eosin stained cross-sections from mice of each genotype sacrificed on the indicated DSS treatment time point at 10x magnification (scale bar = 200 um). Images captured with a Leica digital brightfield microscope and the Leica application software (LAS). (b) Submucosal swelling of the rectal longitudinal folds (dashed line) in the images presented in panel (a) was measured (area *μ*m^2^) using ImageProPlus software. The untreated wild-type rectal section illustrates the region measured (yellow).

Histological analysis, utilizing a scoring system described previously [22, 23], is a valuable tool to semi-quantitatively assess the damage in the colon tissue during experimentally induced colitis events as it is to assess the extent of tissue injury observed in tissues surgically removed from IBD patients. Hematoxylin and eosin (H&E) stained rectal sections (the site where colitis begins in this model) from mice sacrificed at each time point were observed and scored for epithelial erosion/crypt damage, submucosal swelling, leukocyte infiltration, muscularis mucosae hyperplasia, and increased angiogenesis using the point system described in the methods. Representative tissue sections are presented in Figure 6. As the length of time mice exposed to DSS increased, the average colitis scores for all genotype groups also increased (Figure 5(a)). However, a distinct and dramatic separation occurs when comparing the HAS3 null group (colitis score 10.5) to wild-type mice (colitis score 16.5) at day 7 (*P* < 0.005), while there is no difference in the mean score comparison between wild-type and HAS1 null mice at day 7 (Figure 5(b)). The HAS1/3 null group score is lower than the wild-type group score and approaches but does not fully meet statistical significance at this day 7 time point. At day 10 time point, the difference is even more pronounced and demonstrates the decrease in colitis severity when the HAS3 enzyme is absent, both in the HAS3 null and the HAS1/3 null groups (Figure 5(a)).

**Figure 7:**
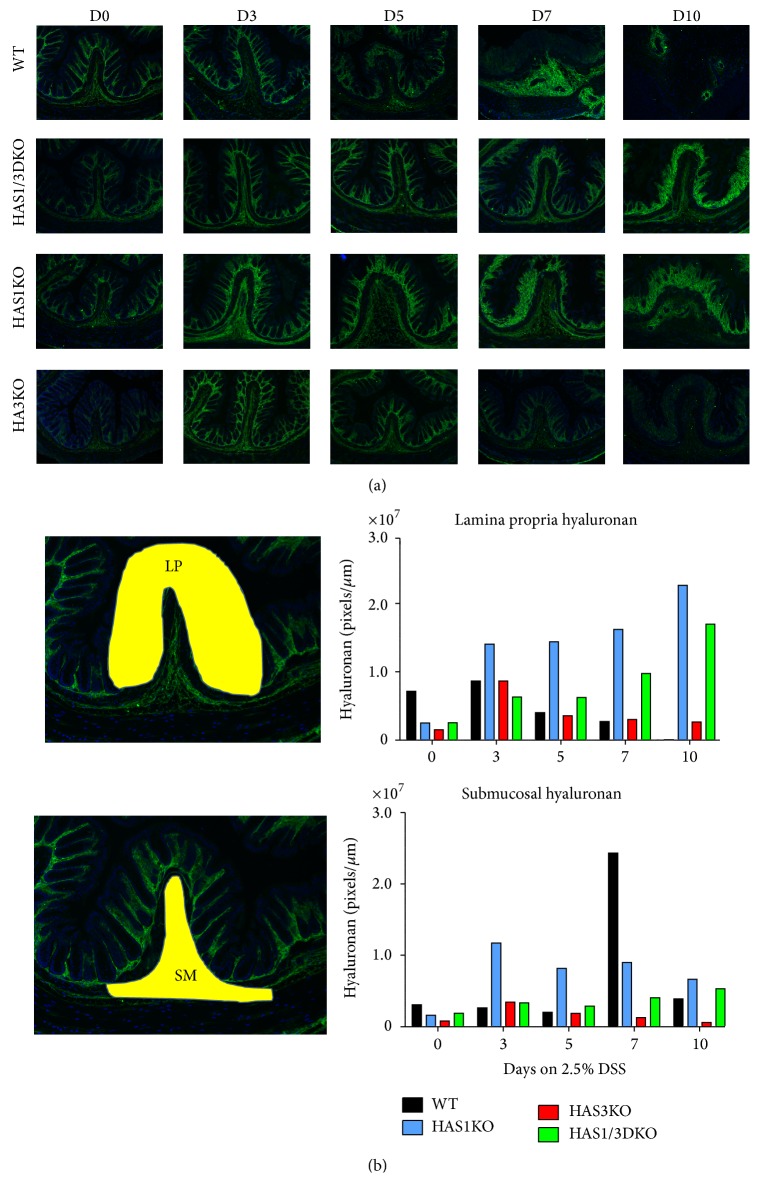
Immunohistochemistry of DSS-treated mice. (a) Adjacent rectal sections to the H&E stained tissues presented in Figure 6 were stained for hyaluronan (green) with the biotinylated hyaluronan binding protein (HABP) and Alexa Fluor streptavidin-488. Nuclei were stained with DAPI (blue). Images are 10x magnification captured with a Leica digital fluorescent microscope and ImageProPlus software. (b) Semiquantitative densitometric analysis of HA staining in longitudinal folds in the images presented in panel (a). The untreated wild-type rectal section illustrates the regions measured (yellow). Lamina propria HA staining (LP) is the region above the muscularis mucosae while submucosa HA staining (SM) is the area below the muscularis mucosae and above the muscularis externa.

The profound changes that occur in the mouse distal colon over the 10 days of DSS-induced colitis can be seen in Figure 6. Distal colon (rectal) sections were selected for specific analysis since this model is bacterially driven and the rectal area has the highest microbe burden. Once the epithelial layers have been effaced and crypt damage ensues due to continuous chemical exposure, direct microbe interaction with host immune cells occurs. Similar pathological changes are routinely observed in human IBD patient intestinal tissue after repeated flares of inflammation. While submucosal swelling and epithelial damage commence at day 3 in wild-type and the HAS1 null groups, this effect is delayed or dramatically reduced in both HAS1/3 and HAS3 null mouse groups even at 10 days of treatment (Figure 6). In addition, leukocyte infiltration, muscularis mucosae hyperplasia, and increased angiogenesis are all evident in day 7 and day 10 in the wild-type and HAS1 null groups but not in HAS1/3 or HAS3 null mice. To determine the extent of submucosal expansion in the longitudinal rectal folds in each tissue over the course of the experimental colitis in Figure 6(a), we measured the area of swelling using imaging software (Figure 6(b)). The representative submucosal region measured is indicated in the untreated wild-type mouse. Both the H&E images and the expansion measurements indicate the dramatic changes in the submucosal region of wild-type and HAS1 null mice, especially at day 7 and day 10, compared to the HAS3 null and HAS1/3 null mice. Immunostaining of these adjacent matched rectal sections for hyaluronan deposition and clearance over the 10-day time course also reveals distinctly different HA staining patterns in the mice lacking the intact HAS3 allele.  Figure 7 demonstrates that in all groups there is increased deposition of hyaluronan at day 3 in the submucosa and mucosal/lamina propria regions of the colon. By day 5, in the wild-type and HAS1 null groups, submucosal swelling is very pronounced, whereas this swelling did not occur in animals with HAS3 deletion. At days 7 and 10, the mice that possess HAS3 (the wild-type and HAS1 null groups) exhibit a very noticeable persistence and increase in hyaluronan deposition in the damaged mucosa as well as around the highly vascularized areas in the submucosa. Semiquantitative densitometric analysis of the HA staining in the longitudinal folds of the lamina propria and submucosal regions was performed to track the changes in hyaluronan in both compartments in each genotype over the course of the colitis (Figure 7(b)). The regions measured for each subcompartment of the rectal fold are illustrated as yellow masked areas on the image from the untreated wild-type mouse. In unchallenged animals, homeostatic levels of HA in the lamina propria extracellular matrix of distal colon tissue are typically decreased with deletion of enzymes HAS1 (~50%) and especially HAS3 (~75%) compared to wild type. In the submucosa, overall lower levels of HA are produced per area, but the relationship of decreased levels of HA with deletion of HAS1 and HAS3 is also observed. Unexpectedly, HA levels in the HAS3 null tissue are lower than in the HAS1/HAS3 double deletion intestinal tissue.

Similar HA analysis was performed on sections obtained from mice undergoing DSS treatment. With DSS challenge, wild-type HA levels in the lamina propria remain relatively constant until day 5 when the HA levels decrease, corresponding to the time that leukocyte infiltration and crypt destruction are evident. Paradoxically, HAS1 null mice have the highest levels of HA at day 3 (1.8-fold compared to wild-type mice) and the higher HA level is maintained throughout the course of disease, reaching 5-fold wild-type levels at day 7 when the disease has reached its pathologic peak. In comparison, HAS3 null animals maintain near wild-type mice levels of HA in the lamina propria throughout the time course, and the HAS1/HAS3 double null animals showed intermediate expression compared to HAS1 null and HAS3 null tissue. The HA content of submucosal tissue also changes during DSS colitis, with wild-type animals showing a great increase in distal colon connective tissue HA at 7 days, the time point when the submucosal inflammatory infiltrate is most evident (Figure 6(a)). Similar to the lamina propria, the HAS1 mice show higher submucosal expression throughout the DSS time course, and the HAS3 mice exhibit normal to low expression. Of note, neither mutant genotype nor the double null peaked at day 7 or exhibited the submucosal inflammation of the wild-type mouse colon. This data provides snapshots of the presence of HA in intestine and how it changes owing to HAS enzyme mutation. However, a causal relationship between HAS expression and HA deposition from this data is not possible to assess since in the inflammation model the cellular makeup of the tissue changes drastically during the disease. Independent of HAS expression, loss of epithelium leads to HA loss from the lamina propria (especially evident on day 10, Figure 6(a)) and the infiltrating leukocytes and platelets have the ability to degrade HA. Additionally, reactive oxygen species generated during inflammation can mediate depolymerization of HA.

**Figure 8:**
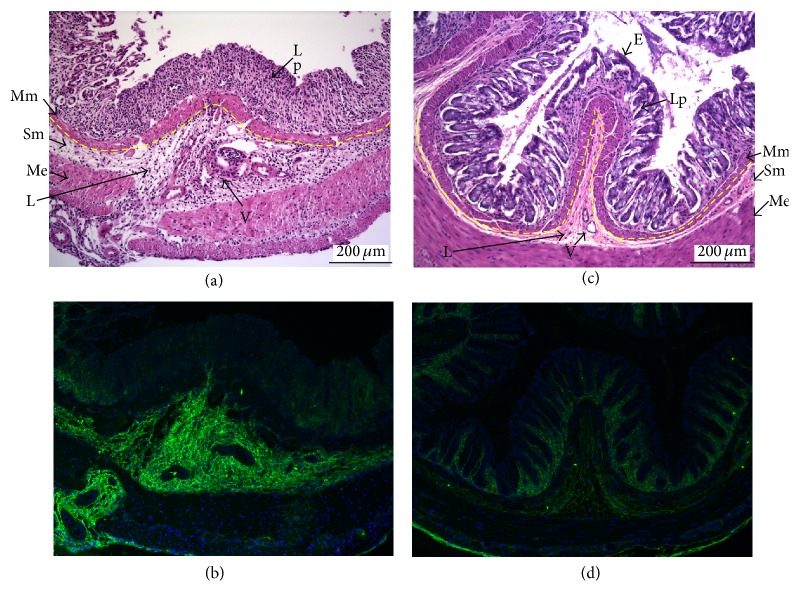
Histology and immunohistochemistry of day 7 DSS-treated wild-type and HAS3 null rectal sections. Cross-section images from Figures 6 and 7 were enlarged to show detail with the following tissue regions marked as follows: (E) epithelium, (Lp) lamina propria, (Mm) muscularis mucosae, (Sm) submucosae, (V) blood vessels, (Me) muscularis externa, and (L) leukocytes. The luminal muscularis mucosa and submucosae boundary are marked in yellow dashed line.

Enlargements of the images from 7-day DSS treatment of wild-type and HAS3 null mice are presented in Figure 8 and they display strikingly different tissue structure. Firstly, observing from the intestinal lumen downward, the lamina propria (Lp) of wild-type animals has lost all epithelium (E) as well as crypt architecture, and infiltrating leukocytes are prevalent in this tissue area (Figure 8(a)). In the HAS3 null section (Figure 8(c)), the epithelium and crypt architecture are largely maintained in the lamina propria, and very little increase in the population of infiltrating leukocytes is noted. Secondly, while the muscularis mucosa (Mm) is slightly expanded in the HAS3 null animals, it does not achieve the overall thickness observed in the wild-type group. Thirdly, the submucosa (Sm) of the wild-type mouse colon is vastly expanded compared to the HAS3 null tissue and contains many more and thicker walled blood vessels (V). Strikingly, there is also a much greater presence of infiltrating leukocytes (L) in the submucosal space of the wild-type mouse colon than in the HAS3 null tissue (Figure 8(a) versus Figure 8(c)). HA deposition within the inflamed and highly vascularized submucosa region of the wild-type sections is tremendously increased compared to either day 7 treated HAS3 null section (Figure 8(b) versus Figure 8(d)) or the untreated wild-type control (Figure 7). The absence of numerous expanded blood vessels and reduced hyaluronan deposition in the HAS3 null submucosa may explain why a leukocyte infiltrate is minor in these mice at day 7.

## 4. Discussion

Hyaluronan can be found in almost every tissue location in the vertebrate body. An adult human turns over an estimated 15 grams of HA every day through balanced tissue specific synthesis by hyaluronan synthase enzymes (HAS1, HAS2, and HAS3) and controlled degradation by hyaluronidase enzymes (HYAL1, HYAL2) [28]. Misregulated HA metabolism has serious physiological consequences in human patients and can be recapitulated in knockout mouse models. Altered HAS enzymes function has been implicated in multiple myeloma (HAS1) [29], in renal-controlled fluid balance, heart formation, long bone/spine development, wound healing (HAS2) [3, 6, 9, 30–33], and seizures (HAS3) [4]. Ablation of hyaluronidase enzyme function results in mucopolysaccharidosis lysosomal storage disorder (HYAL1) [34, 35], craniofacial bone disorders, heart valve defects and thrombocytopenia (HYAL2) [36, 37], and lung carcinoma (HYAL3) [38]. We were therefore surprised that HAS1 null, HAS3 null, and HAS1/HAS3 double null mice had no obvious physical, developmental, or reproductive defects. This finding, together with the data that HAS2 null animals are embryologically lethal, indicates that HAS2 is the crucial HA generator in the body. HAS2 is thought to be the major inducible synthase in mesenchymal cells including fibroblasts and smooth muscle cells [39, 40]. The data presented here showing HA staining of the colon (Figure 7) support this idea. The HA content in unchallenged HAS1/HAS3 null (only HAS2 expressing) colons is not dramatically different than in wild-type mouse tissues that express all three isoenzymes. The question then arises: why are there three enzymes that make the same HA product when HAS2 seems to be sufficient. We hypothesized that HAS1 and HAS3 may play a role during times of stress and tested this notion in a chemically induced, bacterially driven model of inflammation.

In our previous experimental DSS-induced colitis studies and studies reported by others, there is a direct correlation between outward signs of disease and pathological changes that take place in the gut tissues [11, 21]. Interestingly, Zheng et al. have previously reported that both HAS2 and HAS3 mRNA are upregulated in mouse colon tissue during DSS colitis [41]. Histological staining of rectal sections of wild-type mice reveals a very consistent pattern of intestinal damage including epithelial cell and crypt loss; areas of submucosal swelling which contain increased hyaluronan deposition; increased vascularization; and inflammatory cell infiltration into the damaged site. To determine whether perturbing HA production could change the course of colitis, we outbred the HAS1/3 double null mice with wild-type, background matched c57B/6 mice to generate the single HAS1 or HAS3 null strains used in the current DSS-colitis model study. Testing all four genotypes revealed that HAS1 null mice and wild-type mice, both of which retain the HAS3 gene, are equally adversely affected by DSS administration on all points of analysis. The HAS3 null group, similar to the HAS1/3 double null cohort, was far less affected by DSS-induced colitis. In fact, our data indicates that the HAS3 mice are even more resistant that the HAS1/3 group. The HAS3 null mice have the lowest DAI, the lowest colitis score, and the lowest circulating levels of the proinflammatory cytokine IL-6, compared to the wild-type, HAS1 null, and HAS1/3 null groups. Il-6 is cytokine that drives inflammation and is an accepted marker of inflammatory changes, especially in gastrointestinal disorders [42, 43]. Previous reports by others have indicated a strong compensation for the loss of both HAS1 and HAS3 by greater induction of HAS2 [6]. Perhaps this accounts for the small discrepancy between the higher levels of protection observed in the HAS3 null group compared to the HAS1/3 nulls.

Overall, our laboratory focuses on investigating the role of HA in the initiation and perpetuation of the inflammatory cycle observed in patients suffering with IBD. Since we have previously recognized: (1) that HA is present at higher levels in inflamed IBD colon tissue compared to non-inflamed IBD tissue as well as non-IBD controls [10]; (2) that HA can act as a leukocyte adhesion molecule on activated human intestinal smooth muscle cells [17] and microvessel endothelial cells [11]* in vitro*; and (3) that changes in HA remodeling happen prior to inflammation* in vivo* in models of colitis, we asked whether disruption of normal HA production* in vivo* would alter the course of induced intestinal inflammation. In the current work we found that deletion of one of the HA synthesizing enzymes, specifically HAS3, but not HAS1, significantly reduced the course of colitis in mice. Leukocyte infiltration, epithelial loss, blood vessel expansion and tissue damage were all reduced in the absence of HAS3. This suggests that the HA produced by HAS3 expressing cells is critical to mounting an inflammatory response, and that the HAS3 gene is somehow important for responding to challenge. The next question is how does HAS3 accomplish this function? At least three possible explanations are plausible: the HA product is somehow different, the location of expression is different, or the factors that regulate HAS3 gene expression are different, compared to HAS2 especially.

Data supporting the idea that the three HA synthases make different sized products has been put forth; HAS1 and HAS2 in membrane preparations synthesize very large HA chains (average molecular mass 2 × 10^5^~2 × 10^6^ Da) while HAS3 produces a smaller HA size range (1 × 10^5^~1 × 10^6 ^Da) [44]. A possible reason HAS3 appears to contribute so significantly to inflammation may be due to the routine production of smaller sized HA that is known to signal pro-inflammatory, pro-angiogenic responses through Toll-like receptors 4 and 2 [45–47]. However, Brinck and Heldin showed using whole cells expressing the different singular HAS enzymes, that HA size was not substantially different [48]. This differential size distinction theory, in any case, is somewhat difficult to accept in the tissue setting where hyaluronan degrading enzymes and cellular clearance mechanisms are operative and likely to alter all HA sizes even further.

Our previous data provide support that both location of expression and inciting stimulus may be important for the specific HAS3 (versus HAS2) participation in inflammation. We have demonstrated that HAS3 is important for increasing HA on microvascular endothelium under inflammatory conditions (i.e., the presence of TNF*α*), and that the HA produced is leukocyte adhesive [11]. In contrast, intestinal smooth muscle cells produce increased HA, predominantly through regulation of HAS2 (unpublished) and do not produce a leukocyte adhesive matrix in response to TNF*α* [17]. In addition, we have determined that human platelets, when activated, have the ability to fragment the HA on endothelial surfaces using the enzyme HYAL2 [13, 49]. Therefore, our new and previously reported data suggest a scenario in the microvesssels of the intestine to partially explain the pathological changes in DSS colitis and human IBD; the HA produced by HAS3 is both a leukocyte recruitment molecule which aids in extravasation into the intestinal tissue, and additionally is a substrate for platelets to create HA fragments that drive cell activation and angiogenesis. Further experiments using vascular specific deletions of HAS3 may be useful in determining if this scenario is correct.

In addition to the concept that disease progression is due to the HAS gene expression, HA size and location of deposition, one cannot rule out the possible role of the activity of the HAS enzymes as they function to polymerize HA cables. Previous studies have demonstrated that HAS1 and HAS2 enzymes can multimerize into a complex HA generating molecular machine [50, 51]. Inactive HAS2 mutant proteins were shown to dimerize with wild-type HAS2 and consequently blocked HA production. Our data suggests that HAS1 may act as a negative regulator of HA synthesis by HAS2 since both HAS1 null and HAS1/3 double null mutants displayed higher levels of HA deposition during colitis. Definitive confirmation of the role of HAS1 as a spoiler will require the generation of the appropriate tagged enzymes for* in vitro* analysis or the generation of new transgenic mice.

Current regimens utilized to combat inflammation in colitis include anti-TNF [52] and anti-IL-6 [26] therapies. However, some patients stop responding to these therapies. Recently, several groups have reported the use of RNAi therapeutic liposomes [53] or nano-particle molecules [54] to selectively knockdown genes driving angiogenesis in tumor vessels* in vivo*, without causing off-target silencing in other larger blood vessels. Since HAS3 is expressed in the microvasculature, it might one day be possible to selectively target RNAi of HAS3 expression, especially in these patients that no longer respond positively to traditional drug therapies.

## 5. Conclusion

Our data suggest HAS3 plays a crucial role in driving gut inflammation while HAS1 may act to repress the hyaluronan synthesis of the other HAS enzymes. HAS3 null mice exhibit decreased intestinal inflammation and tissue damage in the DSS-induced colitis model compared to wild-type, HAS1 null, and HAS1/3 double null mice while mice lacking HAS1 exhibit heightened HA deposition in both the lamina propria and submucosa during induced colitis. We suggest that future studies aimed at controlling HAS3 expression in the intestinal microvasculature may reveal new therapeutic intervention strategies for patients suffering from IBD.
